# Diterpenoids Isolated from *Podocarpus macrophyllus* Inhibited the Inflammatory Mediators in LPS-Induced HT-29 and RAW 264.7 Cells

**DOI:** 10.3390/molecules26144326

**Published:** 2021-07-17

**Authors:** ChoEen Kim, DucDat Le, Mina Lee

**Affiliations:** 1 College of Pharmacy and Research Institute of Life and Pharmaceutical Sciences, Sunchon National University, 255 Jungangno, Suncheon-si 57922, Jeonnam, Korea; kce313@naver.com (C.K.); ddle@scnu.ac.kr (D.L.); 2 Jeonnam Institute of Natural Resources Research, Jangheung-gun 59338, Jeonnam, Korea

**Keywords:** *Podocarpus macrophyllus*, diterpenoids, RAW 264.7 cells, HT-29 cells, IBD

## Abstract

Species of *Podocarpus* are used traditionally in their native areas for the treatment of fevers, asthma, coughs, cholera, chest pain, arthritis, rheumatism, and sexually transmitted diseases. To identify natural products having efficacy against inflammatory bowel disease (IBD), we identified a new, 16-hydroxy-4β-carboxy-*O*-β-D-glucopyranosyl-19-nor-totarol (**4**) together with three known diterpenoids from *P. macrophyllus*. Furthermore, all the extracts, fractions, and isolates **1**–**4** were investigated for their anti-inflammatory effects by assessing the expression on nitric oxide (NO) production and proinflammatory cytokines in lipopolysaccharide (LPS)-stimulated RAW 264.7 and HT-29 cells. Among them, nagilactone B (**2**) exhibited a potent anti-inflammatory effect against NO production on RAW 264.7 cells; therefore, nagilactone B was further assessed for anti-inflammatory activity. Western blot analysis revealed that nagilactone B significantly decreased the expression of LPS-stimulated protein, inducible nitric oxide synthase (iNOS), cyclooxygenase (COX)-2, and phosphorylated extracellular regulated kinase (pERK)1/2. In addition, nagilactone B downregulated tumor necrosis factor (TNF)-α, interleukin (IL)-6, and IL-8 levels in LPS-induced macrophages and colonic epithelial cells. To our best knowledge, this is the first report on the inhibitory effect of nagilactone B (pure state) and rakanmakilactone G against NO production in LPS-stimulated RAW 264.7 cells. Thus, diterpenoids isolated from *P*. *macrophyllus* could be employed as potential therapeutic phytochemicals for IBD.

## 1. Introduction

Inflammatory bowel disease (IBD) is a complex disease associated with environmental and genetic factors that lead to immunological reactions and inflammation in the intestine [1]. Imbalances in the activation of proinflammatory and anti-inflammatory signaling pathways in the gut can lead to IBD, Crohn′s disease (CD), and ulcerative colitis (UC) as chronic inflammatory conditions of the gastrointestinal system [2]. CD can influence any part of the gastrointestinal tract from the mouth to the perianal area. UC, distinguished by inflammation of the mucosal layer, is limited to the colon [3]. A change in gut microbial composition in genetically susceptible individuals, an altered immune system, and environmental factors are all assumed to play a role in the pathogenesis of IBD [4]. Complications of IBD include diarrhea, chronic inflammation, abdominal pain, cramping, fever, weight loss, wasting, internal bleeding, and ultimately, cancer. IBD prevalence has increased during the past decades, especially in Westernized countries with a prevalence as high as 1%. It is well-known that its prognosis is poor and that its medication is often ineffective [5].

Inflammation is a crucial biological response of the body against various stimuli, leading to the release of proinflammatory cytokines and mediators [6]. It is commonly accepted that acute inflammatory responses are necessary for normal wound repair, while chronic or excessive inflammation may lead to pathological scarring and fibrosis [7]. Nuclear factor-kappa B (NF-*κ*B) is a key transcription factor of classically activated (M1) macrophages. It is involved in the regulation of a number of inflammatory genes including those encoding TNF-α, IL-1β, IL-6, and COX-2 [8]. COX-2 and inducible iNOS are also essential constituents of the inflammatory responses that ultimately repair injury or lead to carcinogenesis [9]. TNF-α is a proinflammatory cytokine with antimicrobial and immunomodulatory features. It is mainly involved in the activation of endothelial and immune cells. NO and TNF-α are mainly produced by activated mononuclear phagocytes, macrophages, and monocytes [10]. Human cyclooxygenase-2 is a highly inducible enzyme that can regulate inflammation, differentiation, mitogenesis, and angiogenesis. NO is a short-lived gaseous molecule that influences the function of many cells [11]. NO is released by endothelial cells in response to relaxation stimuli to reduce the contraction of vascular smooth muscle cells, finally decreasing peripheral blood pressure [12]. On the other hand, mitogen-activated protein kinases (MAPKs) including extracellular signal-regulated kinase (ERK) play pivotal roles in the control of cell differentiation and growth. Their phosphorylation is known to be a critical component in the production of NO and proinflammatory cytokines in activated macrophages [13]. Many studies have focused on finding safe candidate materials for preventing and treating inflammatory diseases through diverse inhibitory actions against upstream signaling events involved in the expression of inflammatory genes.

Some species in the genus *Podocarpus* are used traditionally in their native areas for treating fevers, asthma, coughs, cholera, chest pain, arthritis, rheumatism, and sexually transmitted diseases [14]. *Podocarpus macrophyllus* is native to southern Japan and southern and eastern China. Flavonoids, bioflavonoids, and norditerpenoids have been obtained from its leaves. Norditerpenes and terpenophenolic compounds possess cytotoxic activities [15]. Meanwhile, diterpenoids, biflavonoids, and totarol have been reported to possess antioxidant and anti-inflammatory activities [14]. Especially, bioflavonoids isolated from *P. macrophyllus* are known to modulate proinflammatory gene expression in both in vitro and in vivo studies. They also possess phospholipase A2 and COX-2 inhibitory activities [16]. To find active constituents for treating human diseases from natural sources, we investigated the chemical constituents of *P. macrophyllus* and evaluated their biological effects, focusing on their anti-inflammatory activities.

## 2. Results

### 2.1. Bioactivity-Guided Isolation of Constituents from the Leaves of P. macrophyllus

In this study, dried leaves and twigs of *P. macrophyllus* were extracted with methanol to obtain methanolic extracts. Firstly, these extracts were tested for antioxidant activities using our previous method [17]. DPPH radical-scavenging results revealed that its leaf extract showed stronger antioxidant effect (IC_50_: 24.7 µg/mL) than its twig extract (IC_50_: 58.2 µg/mL). Both extracts displayed similar ABTS radical-scavenging effects, with IC_50_ values around 36 µg/mL (Table 1). These results suggest that the extract of *P. macrophyllus* leaves could be employed for further experiment.

**Figure 1:**
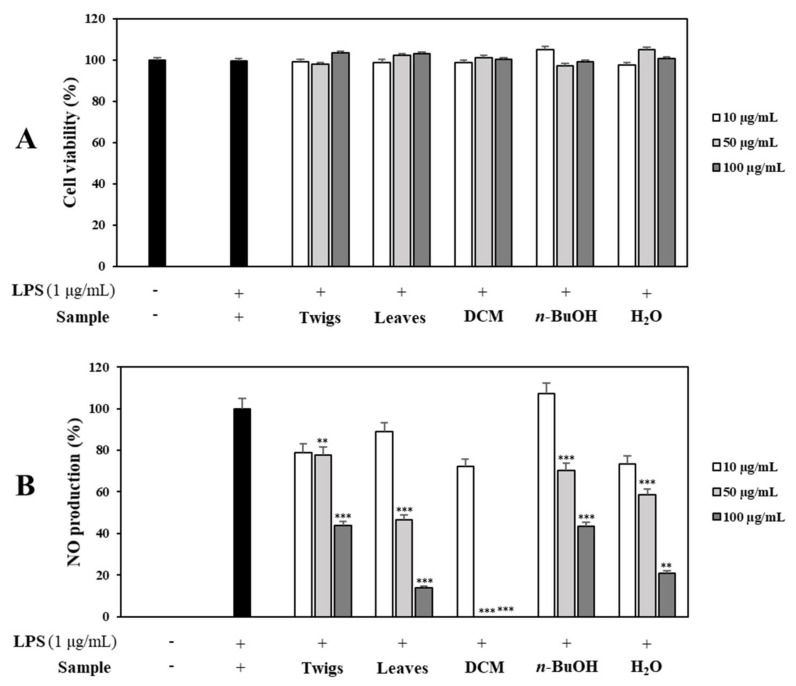
Effects of the total extract and fractions of aerial parts of *P. macrophyllus* on cell viability and NO production. (**A**) RAW 264.7 cells were cultured in the presence of the total extract and extraction fractions (10, 50, and 100 µg/mL) for 1 h and induced with LPS (1 µg/mL) for 16 h. Cell viability and NO production were measured using MTT assay and Griess reagent, respectively. (**B**) Nitrite concentrations of untreated and LPS-treated controls, respectively. Each experiment was performed in triplicates. The data are represented as mean ± SD. ** *p* < 0.01, *** *p* < 0.001 vs. LPS-treated group.

Secondly, both leaf and twig extracts were examined for their NO production inhibitory effects in LPS-stimulated RAW 264.7 cells using different concentrations (10, 50, and 100 µg/mL). Results indicated that its leaf extract exhibited stronger inhibition against NO production than its twig extract. Both leaf and twig extracts showed noncytotoxic effects on cell viability at the same concentrations using MTT assay [18]. Therefore, leaf extract was selected as the material in our research (Figure 1). The leaf extract was suspended in water and successfully partitioned into fractions using CH_2_Cl_2_, *n*-butanol, and water according to solvent polarity. Its crude extract and fractions were tested for their NO production inhibitory effects on RAW 264.7 cells in the presence or absence of LPS-stimulation. None of them showed a cytotoxic effect (cell viability >95%). CH_2_Cl_2_ and *n*-butanol fractions displayed significant concentration-dependent inhibitory effects on NO production. Therefore, these fractions were experimented for further separation using chromatographic methods to obtain four compounds (**1**–**4**).

### 2.2. Structural Elucidation of Isolates ***1**–**4***

**Figure 2:**
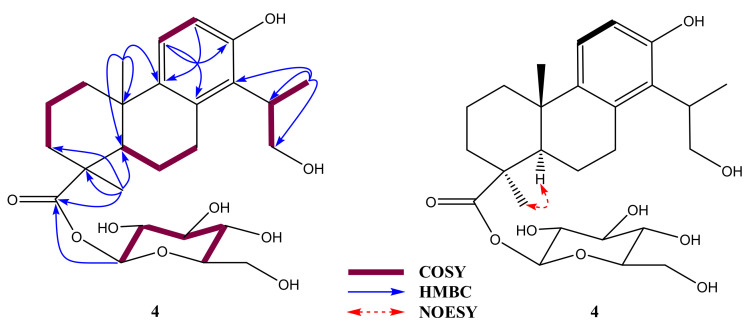
^1^H-^1^H COSY, ^1^H-^13^C HMBC, and ^1^H-^1^H NOSEY correlations of compound **4**.

Compound **4** was obtained as a white amorphous powder. High-resolution electron spray ionization mass (HRESIMS) spectrometry showed a basic ion peak at *m*/*z* 539.2490 [M + HCOOH-H]^−^ (calcd. C_26_H_37_O_9_HCOO, 539.2492), which confirmed eight indices of hydrogen deficiency with a molecular formula of C_26_H_38_O_9_. In addition, the mass spectrum of **4** exhibited the mass transition quantification of *m*/*z* 493.2431 → 331.1908, corresponding to fragmentation reduction [M-H-162]^−^ of a hexose moiety [19]; this indicates that compound **4** contained a pyranose monosaccharide. As shown in Table 2, the unsaturation degree may explain the presence of an aromatic ring, a hexose, and an esterified carboxylic group. The ^1^H-NMR spectrum of **4** indicated the presence of a 2-hydroxy-1-methylethyl moiety, including a secondary methyl proton at *δ*_H_ 1.18 (d, *J* = 6.8 Hz), two oxygenated methylene protons at *δ*_H_ 3.66 (m), and a methine signal at *δ*_H_ 3.09 (m). There were two protons corresponding to an *ortho*-decoupling system at *δ*_H_ 6.53 and 6.91 (d, *J* = 8.2 Hz, H-11/12, each 1H) attributed to an aromatic ring by their COSY correlation and HMBC correlations of H-11 (*δ*_H_ 6.91) to C-8 (*δ*_C_ 134.1)/C-13 (*δ*_C_ 153.3) with H-12 (*δ*_H_ 6.53) to C-9 (*δ*_C_ 138.9). Two methyl singlets were also observed at *δ*_H_ 0.98 (s, H_3_-18) and 1.23 (s, H_3_-20). The HMBC spectrum showed long-range correlations of H_3_-18 (*δ*_H_ 0.98) to C-3 (*δ*_C_ 36.8)/C-5 (*δ*_C_ 51.6)/carboxylic (*δ*_C_ 175.7, C-19) with those of H_3_-20 (*δ*_H_ 0.98) to C-4 (*δ*_C_ 51.6)/C-9 (*δ*_C_ 138.9)/C-10 (*δ*_C_ 38.1). HMBC correlations of H_3_-17 (*δ*_H_ 1.18) to C-14 (*δ*_C_ 127.4)/C-15 (*δ*_C_ 36.0)/C-16 (*δ*_C_ 64.3) propose that 2-hydroxy-1-methylethyl is connected to the aromatic ring through C-14. COSY sequence correlations of H-5/H-6/H-7 were also observed. ^1^H corresponding to ^13^C assignment were accomplished using COSY, HMQC, and HMBC correlations (Figure 2). Spectra of compound **4** are presented in Supplementary Materials (Figures S2.1–S2.9). In addition, an anomeric proton at *δ*_H_ 5.33 (d, *J* = 8.2 Hz, H-1’) together with other aliphatic protons revealed a β-linkage sugar unit. The chemical shift values [*δ*_C_ 93.7 (C-1’), 72.4 (C-2’), 77.4 (C-3’), 69.4 (C-4’), 77.6 (C-5’), and 60.5 (C-6’)] of the sugar unit of **4** were observed and characterized for a D-glucopyranosyl moiety connecting totarol backbone [18,20,21]. The above evidence provide support for a β-D-glucopyranosyl unit in **4**. HMBC correlation of H-1’ to C-19 (*δ*_C_ 175.7) confirmed the sugar location via C-19 position.

These observations approve for a 19-nor-totarol glycoside structure of **4** [18]. Spectroscopic data of **4** were similar to those of 2α,16-dihydroxy-4β-carboxy-*O*-β-D-glucopyranosyl-19-nor-totarol [22] with less than a hydroxyl group. This difference was clarified by COSY sequence correlations of H-1/H-2/H-3 of **4**. Hence, the planar structure of **4** was elucidated to be 16-hydroxy-4-carboxy-*O*-β-D-glucopyranosyl-19-nor-totarol as shown. The relative configuration of **4** was determined based on a comprehensive analysis of its NMR data and NOESY correlations. According to 19-nor-totarol derivative, C-5 and C-10 were assigned as *R*- and *S*-configurations, respectively. Reasonably, the chemical shift value and coupling constant value [*δ*_C_ 51.6 and *δ*_H_ 1.41 (d, *J* = 12.4 Hz)] at C-5 and that of [*δ*_C_ 23.5 and *δ*_H_ 0.99 (s)] at C-20 were similar to those of reported data [18,23,24]. Importantly, the NOESY correlation of H-5 (*δ*_C_ 1.41) to H_3_-18 (*δ*_H_ 1.24) was proposed for CH_3_-18α orientation (Figure 2). With the above information, **4** was named 16-hydroxy-4β-carboxy-*O*-β-D-glucopyranosyl-19-nor-totarol.

**Figure 3:**
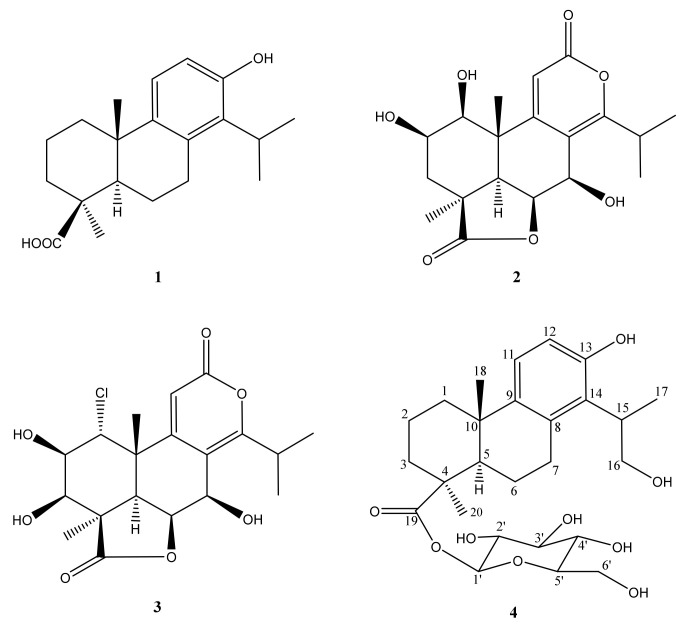
Structures of diterpenoids isolated from *P. macrophyllus*.

In addition, other three known compounds were also isolated and identified as 4β-carboxy-19-nor-totarol (**1**) [18], nagilactone B (**2**), and rakanmakilactone G (**3**) [25] by comparing their spectroscopic and mass data to previous literature (Figure 3).

### 2.3. Biological Evaluation of Isolates ***1**–**4***

#### 2.3.1. NO Production Inhibitory Effect and Cytotoxicity of Isolates **1**–**4**

**Figure 4:**
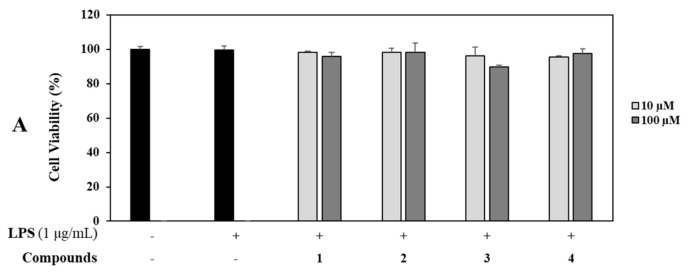

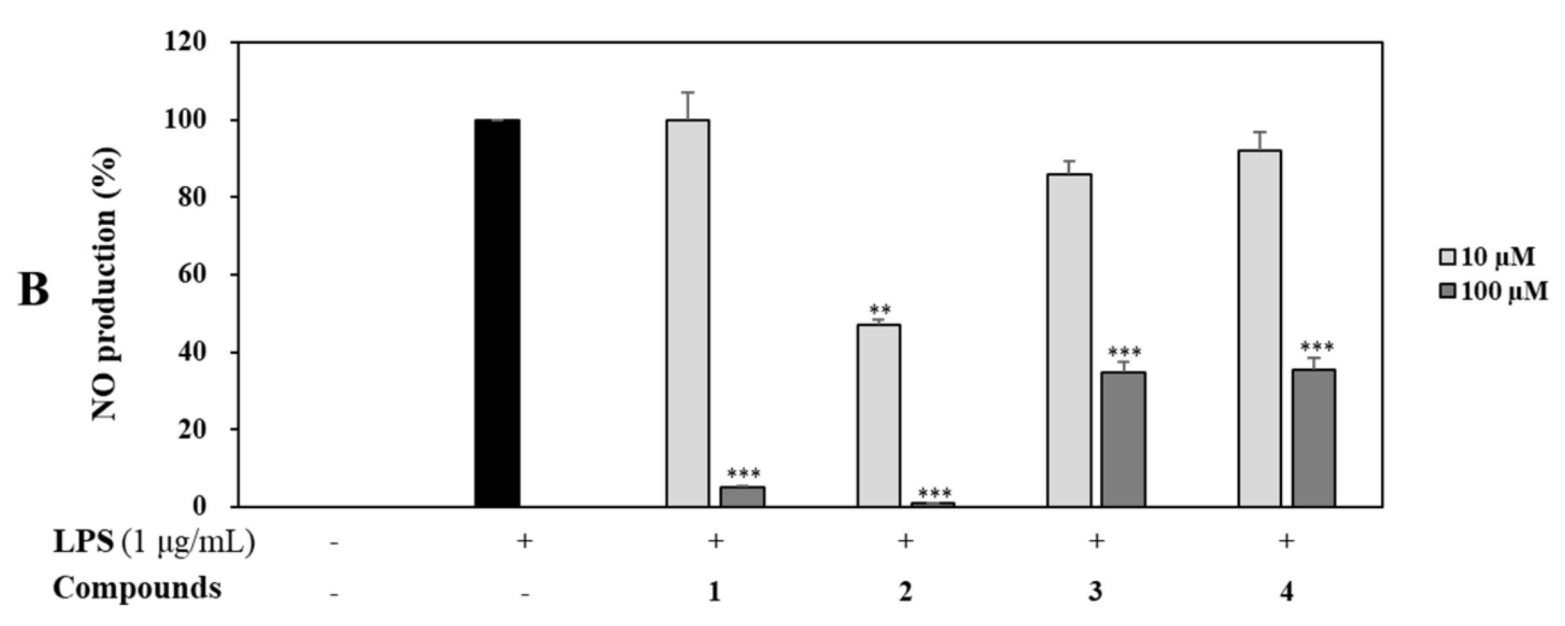
Effects of compounds on cell viability and NO production. (**A**) RAW 264.7 cells were treated with compounds **1**−**4** (10 and 100 µM) for 1 h and stimulated with LPS (1 µg/mL) for 16 h. Cell viability and NO production were detected using MTT assay and Griess reagent, respectively. (**B**) Nitrite concentrations of untreated and LPS-treated controls, respectively. Each experiment was performed in triplicate. The data are represented as mean ± SD. ** *p* < 0.01, *** *p* < 0.001 vs. LPS-treated group.

RAW 264.7 cells were treated with four isolates (**1**–**4**) at concentrations of 10 and 100 µM for 1 h and then induced with LPS (1 µg/mL) for 16 h. Control groups were treated with or without LPS in the absence of sample. The supernatant (100 µL) was harvested and NO production was determined using the Griess assay [26]. Briefly, cells were treated with each compound at the same above concentrations. Cell viability was then determined using MTT assay. As a result, all compounds (**1**–**4**) showed no cytotoxicity. Notably, nagilactone B (**2**) inhibited NO production in LPS-stimulated RAW 264.7 cells most (by 99.1%) at a concentration of 100 µM compared with the control (*p* < 0.001), followed by compound 1 (by 95.0%) and compounds **3** and **4** at the same concentration of 100 µM compared with the control at the same tested concentration (Figure 4).

#### 2.3.2. Effects of Nagilactone B on LPS-Induced iNOS and COX-2 Expression

**Figure 5:**
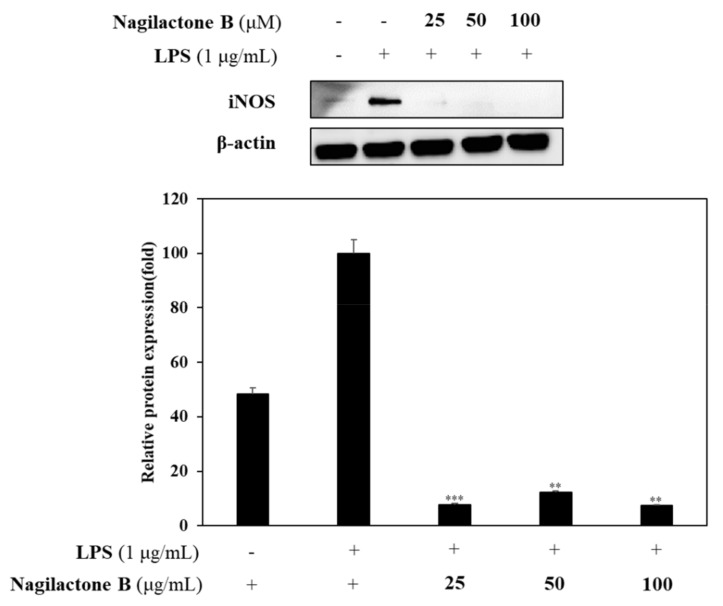

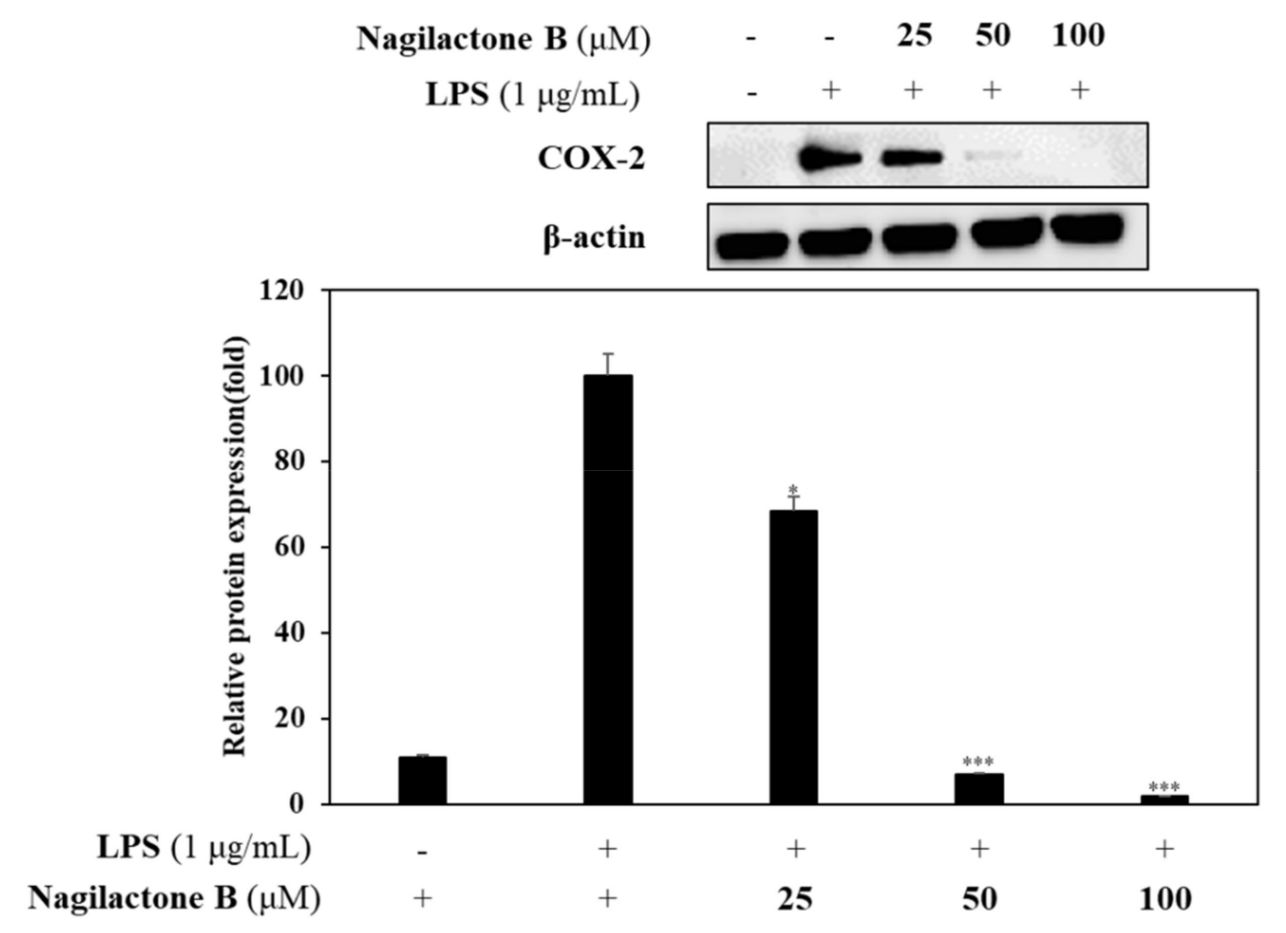
Effects of nagilactone B on iNOS and COX-2 expression. RAW 264.7 cells were incubated in the presence of compounds (25, 50, and 100 µM) for 1 h and induced with LPS (1 µg/mL) for 16 h. The levels of iNOS, COX-2, and β-actin in the LPS-stimulated cells were analyzed by western blot analysis. Relative density was calculated as the ratio of the expression level of each protein with β-actin. * *p* < 0.05, ** *p* < 0.01, *** *p* < 0.001 vs. LPS-treated group.

Western blot analysis was performed to determine the effect of nagilactone B that showed the strongest inhibitory activity on the expression of iNOS and COX-2. As a result, nagilactone B inhibited LPS-induced iNOS expression in comparison with the overexpression of LPS-stimulated mediator without sample pretreatment. Similarly, nagilactone B reduced the expression of LPS-induced COX-2 expression compared to LPS-stimulated COX-2 expression without sample pretreatment. β-Actin was used as an internal control for both experiments (Figure 5).

#### 2.3.3. Effects of Nagilactone B on LPS-Induced pERK 1/2 Expression

**Figure 6:**
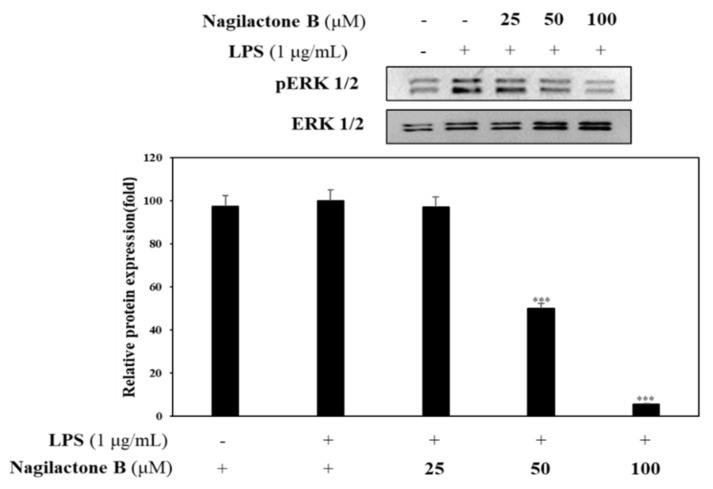
Effect of nagilactone B on pERK 1/2 expression. RAW 264.7 cells were cultured in the presence of compounds (25, 50, and 100 µM) for 1 h and stimulated with LPS (1 µg/mL) for 16 h. The levels of pERK 1/2 were detected by western blot analysis. The results are representative of three independent experiments. Relative density was calculated as the ratio of the expression levels of each protein with β-actin. *** *p* < 0.001 vs. LPS-treated group.

Nagilactone B showed potent inhibition on NO production. Thus, a further experiment was designed to determine phosphorylated ERK expression. RAW 264.7 cells were pretreated with nagilactone B at various concentrations (25, 50, and 100 µM) for 1 h prior to LPS stimulation to investigate the inhibitory effect of nagilactone B on phosphorylated ERK1/2. Results indicate that nagilactone B significantly reduced production of phosphorylated ERK1/2 compared to LPS-stimulated pERK 1/2 without sample pretreatment (Figure 6).

#### 2.3.4. Effects of Nagilactone B on LPS-Induced Proinflammatory Cytokines Production

**Figure 7:**
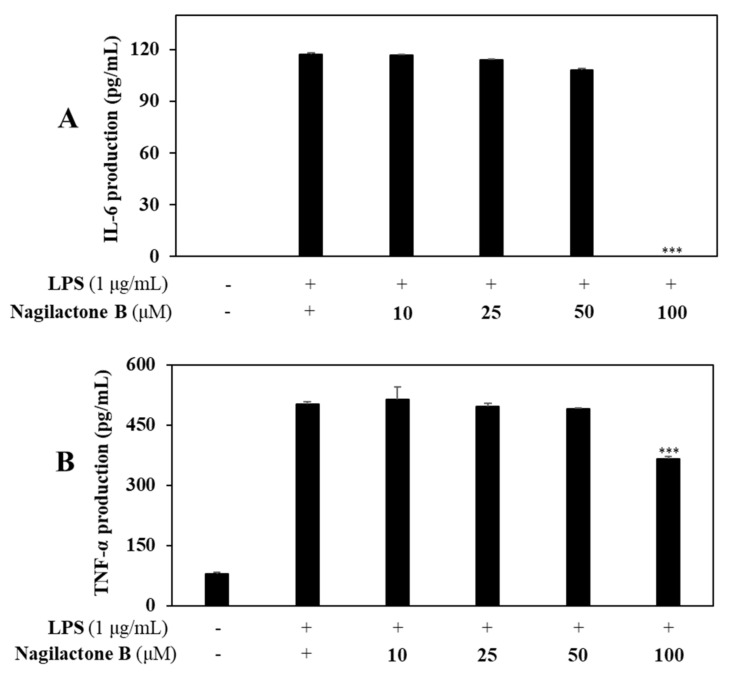
Effect of nagilactone B on the expression of IL-6 and TNF-α. RAW 264.7 cells were treated with nagilactone B (10, 25, 50, and 100 µM). Levels of IL-6 (**A**) and TNF-α (**B**) in the culture media were measured by ELISA. Cell viability was treated with compounds at the same concentrations and detected using MTT assay. The data are expressed as the mean ± SD (*n* = 3). *** *p* < 0.001 vs. LPS-treated group.

To determine whether nagilactone B, which showed the strongest inhibitory effect on NO production, might inhibit inflammatory response, experiments were designed using RAW 264.7. Levels of IL-6 and (TNF)-α production were then measured using ELISA kits. Controls were carried out with or without LPS-stimulation of RAW 264.7 cells in the absence of sample pretreatment. As a result, nagilactone B inhibited the production of IL-6, and (TNF)-α by 99.3% and 28.1%, respectively, compared with controls (Figure 7).

**Figure 8:**
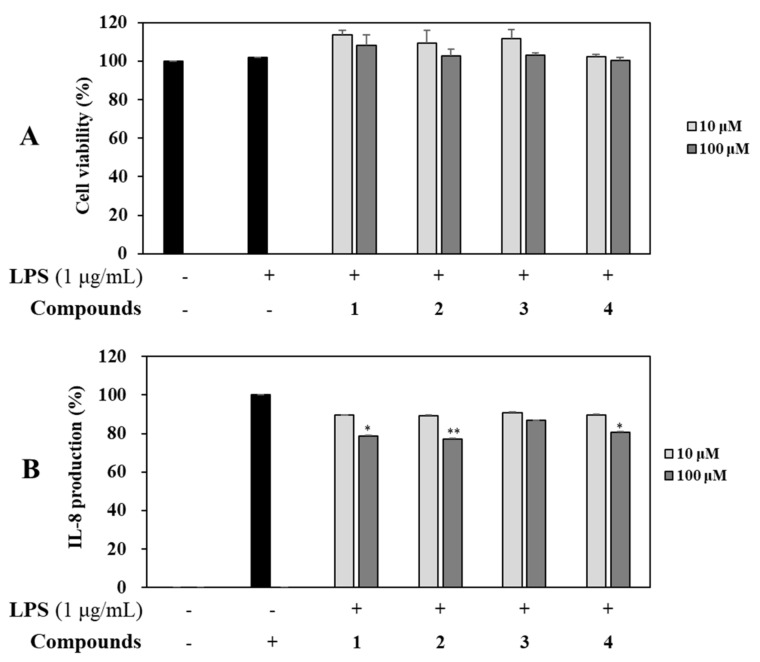
Effects of isolates **1**–**4** on the LPS-induced expression of IL-8 in HT-29 cells. HT-29 cells were treated with nagilactone B (10 and 100 µM) for 2 h and stimulated with 100 ng/mL LPS for 18 h. The viability of cells was then determined using an MTT assay (**A**) and the level of IL-8 in the culture media was measured with an ELISA kit (**B**). The values are expressed as mean ± standard deviation of three individual experiments. Compared with the control group; * *p* < 0.05, ** *p* < 0.01 vs. LPS-treated group.

Additionally, effects of all isolated compounds **1**–**4** on the production of inflammatory mediator (IL-8) were determined at concentrations of 10 and 100 µM using ELISA kits. Compared to the untreated control group, levels of IL-8 were increased in LPS-stimulated colon epithelial cells. Among the four compounds, rakanmakilactone G (**3**) resulted in the highest level of IL-8, followed by compounds **4**, **1**, and **2**. All these compounds showed noncytotoxic effects based on cell viability results using MTT assay (Figure 8).

## 3. Discussion

Initiation and inducibility of inflammatory mediators; high expression of inflammatory cytokines including TNF-α, IL-6, and chemokines (CXC chemokine receptor 4); and transformation in the expression of specific microRNAs may play a role in oxidative-stress-induced inflammation [27]. This inflammatory/oxidative environment can trigger an unhealthy circle that affects healthy stromal and neighboring epithelial cells, which may trigger carcinogenesis after a long period of time [28].

Antioxidant and anti-inflammatory effects in the primary studies from the crude extracts from leaves and twigs of *P. macrophyllus* result in the selection of raw material in our study. Subsequently, the most potential fractions from leaf methanolic extract were experimented leading to the isolation of a new (**4**) and three known compounds (**1**–**3**). These isolates displayed potent inhibition against NO production in LPS-stimulated RAW 264.7 cells. Particularly, nagilactone B (**2**) showed the most potent anti-inflammatory effect than those of **1**, **3**, and **4** at the same tested concentration. A mixture of 2-epinagilactone B and nagilactone B was examined for anti-inflammatory effect on LPS-induced NO production without signaling factor involved inhibition in previous study [29]. In our experiment, nagilactone B was obtained in its pure state (see Figures S1.1 and S1.2, Supplementary Materials) and showed potent inhibition against NO production in LPS-stimulated RAW 264.7 cells at a concentration of 100 μM (Figure 4). Notably, nagilactone B and rakanmakilactone G possessed the same nagilactone skeleton. However, nagilactone B exhibited a stronger inhibitory effect than rakanmakilactone G, suggesting that the presence of OH-3 group in rakanmakilactone G and Cl-1 group in rakanmakilactone G instead of OH-1 in nagilactone B are not favorable for inhibiting NO production in our experiment. In the same manner, 4β-carboxy-19-nor-totarol (**1**) and 16-hydroxy-4β-carboxy-*O*-β-d-glucopyranosyl-19-nor-totarol (**4**) were derived from the same nor-totarane diterpene skeleton. However, 4β-carboxy-19-nor-totarol inhibited NO production more strongly than 16-hydroxy-4β-carboxy-*O*-β-d-glucopyranosyl-19-nor-totarol. This indicates that the presence of sugar unit may reduce the inhibitory effect.

Nitric oxide radical is induced during inflammatory produce and generated via oxygen and l-arginine using iNOS and controlled inflammatory process [30]. As a similar inflammatory mediator, COX-2 is related to regulation in response to processing of infections, atherosclerosis, and cancers. It catalyzes the step in prostaglandin (PG) biosynthesis overstimulation of cyclooxygenase and sequence PG synthase activity on arachidonic acid. It has been demonstrated that iNOS and COX-2 play pivotal roles in tumors and inflammatory diseases [31].

The most active compound, nagilactone B, was performed to confirm its anti-inflammatory activity. Nagilactone B significantly reduced the expression of both iNOS and COX-2 enzymes in LPS-stimulation or the downregulated inflammatory process. Thus, nagilactone B might be considered as a candidate for inflammatory chemoprevention.

Previously, it has been shown that NO levels can affect the gene network containing ERK dual specific MAP kinase phosphatases and downregulate ERK activity. The expression of some genes associated with development is influenced by ERK phosphorylation [32]. Nagilactone B downregulated the production of phosphorylated ERK1/2. The result confirmed the anti-inflammatory property of nagilactone B.

Mast cells can mediate inflammatory responses and release proinflammatory cytokines, including IL-6, IL-8, and TNF-α, as well as inflammatory mediators (iNOS and COX-2) [33]. In addition, NO activation may initiate the transcription of downstream inflammatory cytokines that can stimulate enzyme mediators and affect signaling intermediation of MAPK pathways [31,34].

Our efforts to investigate the inhibitory effects of isolated compounds on LPS-induced IL-6, IL-8, and TNF-α production. Among them, nagilactone B strongly inhibited production levels of IL-6, IL-8, and TNF-α in LPS-stimulated RAW 264.7 cells; additionally, nagilactone B also exhibited a stronger inhibitory effect than those of other isolates in LPS-stimulated colon epithelial cells. Thus, nagilactone B might inhibit inflammatory response in the current experimental conditions.

## 4. Materials and Methods

### 4.1. General Procedures and Chemical Regents

The NMR spectra of isolates **1**–**4** were recorded on a JEOL JNM-AL 400 MHz spectrometer (JEOL, Tokyo, Japan), and chemical shifts were expressed as *δ* values (ppm) with TMS as the internal standard (measured in DMSO-*d*_6_). The mass confirmation was performed on a Thermo scientific Vanquish UHPLC system (Thermo Fisher Scientific, Sunnyvale, CA, USA) coupled with Shimadzu column and a triple TOF 5600+ mass spectrometer system (Triple ToF MS) (SCIEX Foster City, CA, USA). Column chromatography (CC) was performed on silica gel (Kieselgel 60, 70–230 mesh and 230–400 mesh, Merck, Darmstadt, Germany), thin layer chromatography (TLC) using precoated silica gel 60 F_254_ (1.05554.0001, Merck) and RP-18 F_254S_ plates (1.15685.0001, Merck) and visualized by spraying with aqueous 10% H_2_SO_4_ and heating for 1.5–2 min. HPLC was performed using a Waters 2695 with UV 996 Photodiode array detector and YMC Pak ODS-A column (20 × 250 mm, 5 µm particle size, YMC Co., Ltd., Kyoto, Japan). HPLC solvents were purchased from Burdick & Jackson, Muskegon, MI, USA and Fisher Chem Alert Guide, Nazareth, PA, USA. All other chemicals and solvents of analytical grade were purchased and used without purification. Dulbecco’s modifie3103d Eagles medium (DMEM), fetal bovine serum (FBS), penicillin, and streptomycin were purchased from Hyclone (HyClone, Logan, UT, USA). Bacterial LPS (Escherichia coli 0111: B4) was purchased from Sigma-Aldrich (St. Louis, MO, USA). Enzyme-linked immunosorbent assay (ELISA) kits for IL-6, and TNF-α were purchased from BD Biosciences (San Diego, CA, USA). A protein-extraction solution was purchased from IntRon (Sungnam, Korea). Antibodies such as ERK 1/2, pERK 1/2, NF-*κ*B p65, and β-actin were purchased from Santa Cruz Biotechnology (Santa Cruz, CA, USA). SuperSignal chemiluminescent substrate was purchased from Thermo Fisher Scientific (Waltham, MA, USA).

### 4.2. Plant Materials

The *P. macrophyllus* twigs and leaves were collected from Seoul National University Forests (Gwangyang, Korea) in September 2019 and identified by Professor Lee, Mina (College of Pharmacy, Sunchon National University). A voucher specimen (SCNUP25) was deposited at the laboratory of pharmacognosy, college of pharmacy, Sunchon National University (Suncheon, Korea).

### 4.3. Extraction and Isolation

The leaves of *P. macrophyllus* (457.1 g) were dried, smashed, then extracted three times with 100% methanol (4 L) for 3 h using ultrasonication. The residue was concentrated in vacuo to yield crude extract (89.1 g). The extract was suspended in H_2_O and fractioned in regular sequence with dichloromethane (DCM) (4 L), *n*-butanol (BuOH) (4 L), and H_2_O to obtain residues of 22.0 g, 17.4 g, and 23.0 g, respectively. Among them, the DCM and BuOH fractions showed significant inhibition effects on NO production in LPS-induced RAW 264.7 cells. These fractions were used for further isolation work. The DCM fraction was used for silica gel column chromatography using a gradient solvent (*n*-hexane:ethylacetate = 9:1 → 100% MeOH) to obtain fifteen fractions (D1–D15). The D4 subfraction yielded compound **1** (t_R_ 13.57, 4.2 mg) when subjected to reverse phase high-performance liquid chromatography (RP HPLC) (YMC-Triart, C_18_ column, 250 × 10 mm, CH_3_CN:H_2_O = 80:20 → 100:0). The BuOH fraction was separated with an open LC system (silica gel, CHCl_3_:MeOH:H_2_O = 400:4:1 → 100% MeOH) and afforded twenty-three subfractions (B1–23). The B6 subfraction yielded compounds **2** (t_R_ 11.21, 1.1 mg) and **3** (t_R_ 18.24, 0.4 mg) when subjected to RP HPLC (YMC-Triart, C_18_ column, 250 × 10 mm, CH_3_CN:H_2_O = 30:70→ 100:0). Compound **4** (t_R_ 35.84, 0.9mg) was isolated from the B10 and B11 subfraction by RP HPLC (YMC-Triart, C_18_ column, 250 × 10 mm, CH_3_CN:H_2_O 25:75 → 100:0), respectively, 16-hydroxy-4β-carboxy-*O*-β-d-glucopyranosyl-19-nor-totarol (**4**).

Whitish amorphous powder; chemical formula: C_27_H_39_O_11_; ^1^H-NMR (400 MHz, DMSO-*d*_6_) and ^13^C-NMR (100 MHz, DMSO-*d*_6_), see Table 2; High-resolution electrospray ionization mass spectrometry (HRESIMS) (negative mode) *m*/*z* 539.2490 [M+HCOOH-H]^−^ (calcd. C_27_H_39_O_11_, 539.2498); ESI-MS/MS (-) *m*/*z* 493.2431 [M-H]^−^, 331.1908 [M-H-Glc]^−^.

### 4.4. ABTS and DPPH Radicals Scavenging Assay

The antioxidant assay of extracts and compounds was performed by 2,2-azino-*bis* (3-ethylbenzothiazoline-6-sulfonic acid diammonium salt (ABTS) and 1,1-diphenyl-β-picrylhydrazine (DPPH) (Sigma-Aldrich, St. Louis, MO, USA) radical scavenging activities, which were measured as described previously [17].

### 4.5. Cell Culture and Cell Viability

Mouse macrophage (RAW 264.7) and human colonic epithelial cells (HT-29) were acquired from Korean Cell lines bank (Seoul, Korea). These cells were grown at 37 °C in Dulbecco’s modified Eagle’s medium (DMEM) supplemented with 10% heat-inactivated FBS, streptomycin sulfate (100 μg/mL), and penicillin (100 IU/mL) in a humidified atmosphere of 5% CO_2_. After preincubation of RAW 264.7 and HT-29 cells for 1 h, 10–100 μM of each compound were added. RAW 264.7 and HT-29 cell viabilities after 24 h of continuous exposure to the compounds were measured with a colorimetric assay based on the ability of mitochondria in viable cells to reduce 3-(4,5-dimethylthiazol-2-yl)-2,5-diphenyltetrazolium bromide (MTT) (Sigma-Aldrich, St. Louis, MO, USA). Cells were added with MTT, incubated for 2 h at 37 °C, and treated with 100 μL of DMSO to solubilize a crystal form of formazan. After, the formazan crystal amount was measured using a microplate reader (Bio-Tek Instruments, Inc, Winooski, VT, USA) at 540 nm. Survival of macrophage cells after treatment with compounds was calculated using the following formula: viable cell number (%) = OD_540_ (treated cell culture) × 100/OD_540_ (vehicle control).

### 4.6. NO Assay

The level of NO production was determined by measuring the amount of nitrite from the cell culture supernatants as described previously [35]. Briefly, RAW 264.7 cells (1 × 10^5^ cells/well) were pretreated with various concentrations of four diterpenoids (**1**–**4**) for 1 h and stimulated with or without LPS for 24 h. After stimulation for 24 h, the cell culture supernatant was then incubated with an equal volume of Griess reagent by using equal volumes of 1% (*w*/*v*) sulfanilamide in 5% (*v*/*v*) phosphoric acid and 0.1% (*w*/*v*) *N*-(1-naphtyl) ethylenediamine at room temperature (RT) for 15 min. The absorption was measured at 550 nm by microplate reader (Bio-Tek Instruments, Inc, Winooski, VT, USA).

### 4.7. Enzyme-Linked Immunosorbent Assay (ELISA)

Cells were pretreated with various concentrations of four isolates (**1**–**4**) for 1 h and stimulated with LPS for 24 h. The cultured media of HT-29 cells and RAW 264.7 cells were collected for measuring the levels of IL-8, TNF-α, and IL-6, respectively. Microtiter plates (Costar, Corning, NY, USA) were added with coating buffer (14.2 mM Na_2_CO_3_, 34.9 mM NaHCO_3_, and 3.1 mM NaN_3_; pH 9.6) and incubated overnight at 4 °C. The plates were washed with PBST containing 0.05% Tween-20 (three times) and blocked with PBST containing 1% bovine serum albumin (BSA) for 1 h at 37 °C. The plates were supplemented with 100 μL of sample or standard IL-8 and incubated for 2 h at RT. The plate was washed with PBST and treated with 100 μL of biotinylated goat antimouse IgG and incubated for 1 h at 37 °C. The Streptavidin–horseradish peroxidase (HRP) conjugate (Amersham, Buckinghamshire, UK) was added to the plate. After treatment with 100 μL of tetramethylbenzidine, the plate was incubated for 30 min at RT and color emerged. The reaction was terminated with 4 M H_2_SO_4_, and OD values at 450 nm were obtained using an enzyme-linked immunosorbent assay (ELISA) reader.

### 4.8. Western Blot

Cells were incubated with various concentrations of four isolates (**1**–**4**) for 1 h and stimulated with LPS for 24 h. Proteins were extracted from cells in ice-cold phosphate-buffered saline (PBS), and followed by lysis buffer (50 mM Tris, pH 7.4, 1 mM EDTA, 0.1% Triton X-100, 1 mM PMSF, 25 μg/mL leupeptin, and 20 μg/mL pepstatin) for 20 min. After centrifugation at 12,000× *g* for 20 min, the protein concentration in the supernatant were determined. Protein was separated by sodium dodecyl sulfate (SDS)–polyacrylamide gel electrophoresis (PAGE) and followed by transferring to a polyvinylidene difluoride membrane (Millipore, Bedford, MA, USA). The membrane was blocked with TBST (10 mM Tris (pH 7.4), 100 mM NaCl, and 0.5% Tween 20), containing 5% BSA, for 30 min at RT and incubated with iNOS, COX-2, and ERK 1/2 antibodies (1:1000, Cell Signaling Technology, Inc., Beverly, MA, USA) in TBST containing 5% skim milk powder overnight at 4 °C, then washed three times with a wash buffer [1 M Tris-HCl (Ph 7.4), 4 M NaCl, Tween-20 in DW] for 10 min. They were incubated in specific secondary antibodies (1:2000 dilution) which conjugated antimouse–IgG (1:1000, Santa Cruz, CA, USA). After binding of an appropriate secondary antibody coupled to horseradish peroxidase, proteins were visualized by enhanced chemiluminescence according to the instructions of the manufacturer (ECL-kit, Thermo Fisher Scientifc, Waltham, MA, USA). The membranes were treated with ECL western blot analysis detection reagents and the fluorescence was detected using a bioimaging system. (MicroChemi 4.2 Chemilumineszenz-System, Israel).

### 4.9. Statistical Analysis

Data were represented as the means ± standard deviations (S.D.) of three replicates. The student t-test was used for statistical analyses of the differences noted. *p* < 0.05 was accepted as statistically significant.

## 5. Conclusions

In summary, oxidant and inflammatory screening of crude extracts and fractions guided the isolation of four compounds (**1**–**4**) (one new and three known nor-diterpenoid derivatives) from leaves of *P. macrophyllus*. Among them, 4β-carboxy-19-nor-totarol (**1**) and nagilactone B (**2**) exhibited strong inhibitory activities against NO production in LPS-stimulated RAW 264.7 cells. These active compounds showed potential anti-inflammatory effects without showing side effects of cytotoxicity. It was notable that rakanmakilactone G and nagilactone B (as pure state) were firstly evaluated for its inhibitory effect on LPS-induced NO production in RAW 264.7 cells. In addition, their structure–activity relationships were proposed according to their biological activities. Enzymatic activities of nagilactone B were determined based on its inhibitory effect on NO production. Nagilactone B downregulated the expression of iNOS and COX-2 mediators. The inhibitory effect of nagilactone B was further confirmed based on the production of proinflammatory cytokines and pERK 1/2 expression. Thus, this compound is a potential candidate for treating inflammatory diseases. Results of the current study support phytochemical discovery of abundant secondary metabolites of *P. macrophyllus*. Effects of its active constituents on markers of inflammation were determined by western blot analysis. Our results revealed anti-inflammatory activities of a new compound not reported previously.

## Figures and Tables

**Table 1:** DPPH and ABTS radical-scavenging activities of leaf and twig extracts from *P. macrophyllus*.

Parts	DPPH (IC_50_, μg/mL)	ABTS (IC_50_, μg/mL)
Leaves extract	24.7	36.4
Twigs extract	58.2	36.2
Vitamin C *	24.8	-
BHT *	101.4	34.1

* Control: Vitamin C (DPPH assay) and BHT (Dibutyl hydroxy toluene, ABTS assay). Data are expressed as the mean ± SD (*n* = 3) of three individual experiments. *p* < 0.05, compared to control.

**Table 2:** ^1^H- and ^13^C-NMR data for compound **4** in DMSO-*d*_6_.

No.	*δ*_H_ (mult., *J* in Hz) ^a^	*δ_C_* ^b^
1	1.20 * 2.14 (m)	40.1, CH_2_
2	1.06 (d, 6.6) 2.10 (m)	36.8, CH_2_
3	1.47 * 1.95 (m)	36.8, CH_2_
4	-	43.4, C
5	1.41 (d, 12.4)	51.6, CH
6	1.85 (dd, 4.8, 12.6) 2.95 (dd, 4.2, 16.5)	33.1, CH_2_
7	2.50 *	40.1, CH_2_
8	-	134.0, C
9	-	138.9, C
10	-	37.9
11	6.92 (d, 8.6)	124.0, CH
12	6.54 (d, 8.6)	114.3, CH
13	-	153.3, C
14	-	127.3, C
15	3.09 (m)	35.7, CH
16	3.68 (m)	64.5, CH_2_
17	1.19 (d, 6.9)	15.1, CH_3_
18	1.24 (s)	28.0, CH_3_
19	-	175.3, C
20	0.99 (s)	23.5, CH_3_
*OGlc*	–	
1’	5.34 (d, 8.0)	93.9, CH
2’	3.12 *	72.6, CH
3’	3.20 (m)	76.7, CH
4’	3.13 *	69.6, CH
5’	3.13 *	77.6, CH
6’	3.44 * 3.59 (dd, 4.1, 11.1)	60.7, CH_2_

Assignments were achieved by HMQC, HMBC, and NOESY experiments.* Overlapped signals. ^a^ 400 MHz, ^b^ 100 MHz.

## Data Availability

Not applicable.

## Supplementary Materials

The following are available online. Figures S1.1 and S1.2: 1D-NMR spectra of compound 2, Figures S2.1–S2.9: 1D/2D-NMR spectra of compound **4**.

## Sample Availability

Samples of the compounds **1****−4** are available from the authors.
