# Supplementary material for: Diterpenoids Isolated from Podocarpus macrophyllus Inhibited the Inflammatory Mediators in LPS-Induced HT-29 and RAW 264.7 Cells
Source: Molecules. 2021 Jul 17;26(14):4326. doi: 10.3390/molecules26144326 (PMC8307039; doi:10.3390/molecules26144326)
Supplement: Supplementary file 1 [file molecules-26-04326-s001.zip › molecules-1275205-supplementary.pdf]

molecules

MDPI

Communication

Diterpenoids Isolated from *Podocarpus macrophyllus* Inhibited the Inflammatory Mediators in LPS-Induced HT-29 Cells and RAW 264.7 Cells

ChoEen Kim [1,2,†], DucDat Le [1,†], and Mina Lee [1,*]

[1] College of Pharmacy and Research Institute of Life and Pharmaceutical Sciences, Sunchon National University, 255 Jungangno, Suncheon-si 57922, Jeonnam, Korea; kce313@naver.com(C.K.); ddle@scnu.ac.kr (D.D.L.)
[2] Jeonnam Institute of Natural Resources Research, Jangheung-gun 59338, Jeonnam, Korea
* Correspondence: minalee@sunchon.ac.kr; Tel.: +82-61-750-3764; Fax: +82-61-750-3708
† These authors contributed equally to this work

Table of Contents

1. Spectral data of nagilactone B (2) **1**

Figure S1.1. ^{1}H NMR (DMSO-d_6, 400 MHz) spectrum of **2** 1

Figure S1.2. ^{13}C NMR (DMSO-d_6, 100 MHz) spectrum of **2** 1

2. Spectral data of new compound 4 .... **2**

Figure S2.1. ^{1}H NMR (DMSO-d_6, 400 MHz) spectrum of **4** 2

Figure S2.2. ^{13}C NMR (DMSO-d_6, 100 MHz) spectrum of **4** 2

Figure S2.3. ^{1}H–^{1}H COSY spectrum of **4** 3

Figure S2.4. ^{1}H–^{13}C HMQC spectrum of **4** 3

Figure S2.5. ^{1}H–^{13}C HMBC spectrum of **4** 4

Figure S2.6. DEPT spectrum of **4** 4

Figure S2.7. ^{1}H–^{1}H NOESY spectrum of **4** 5

Figure S2.8. HR-ESI-MS spectrosopic data of **4** 5

Figure S2.9. Extend ESI-MS/MS spectrometry of **4** 6

1. Spectra of nagilactone B (2):

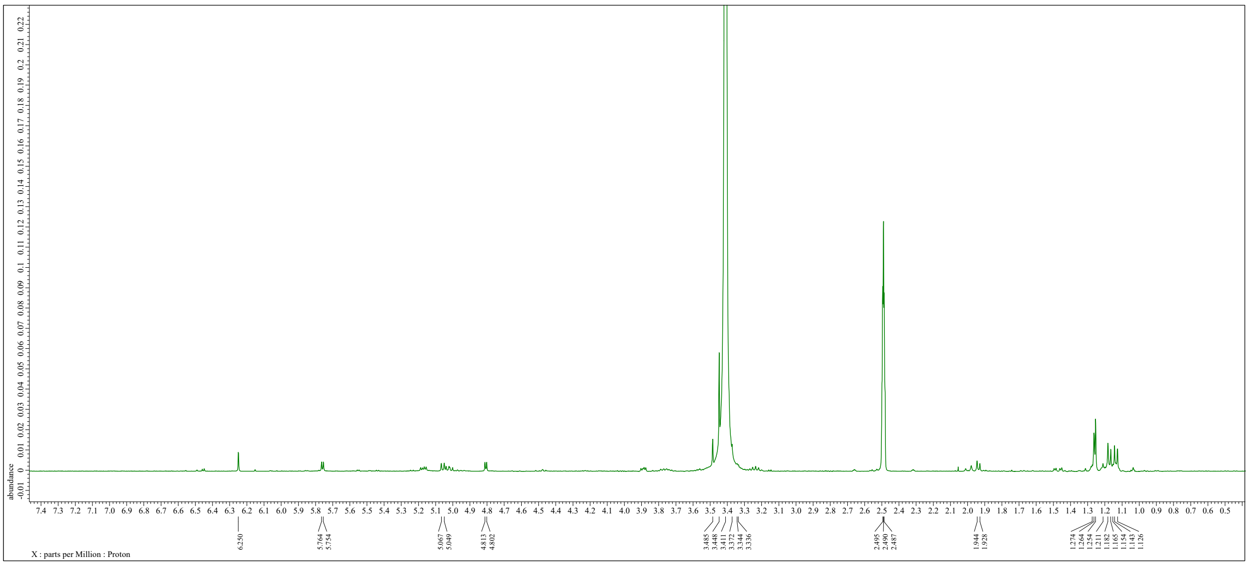

Figure S1.1. ^{1}H NMR (DMSO-d_6, 400 MHz) spectrum of **2**.

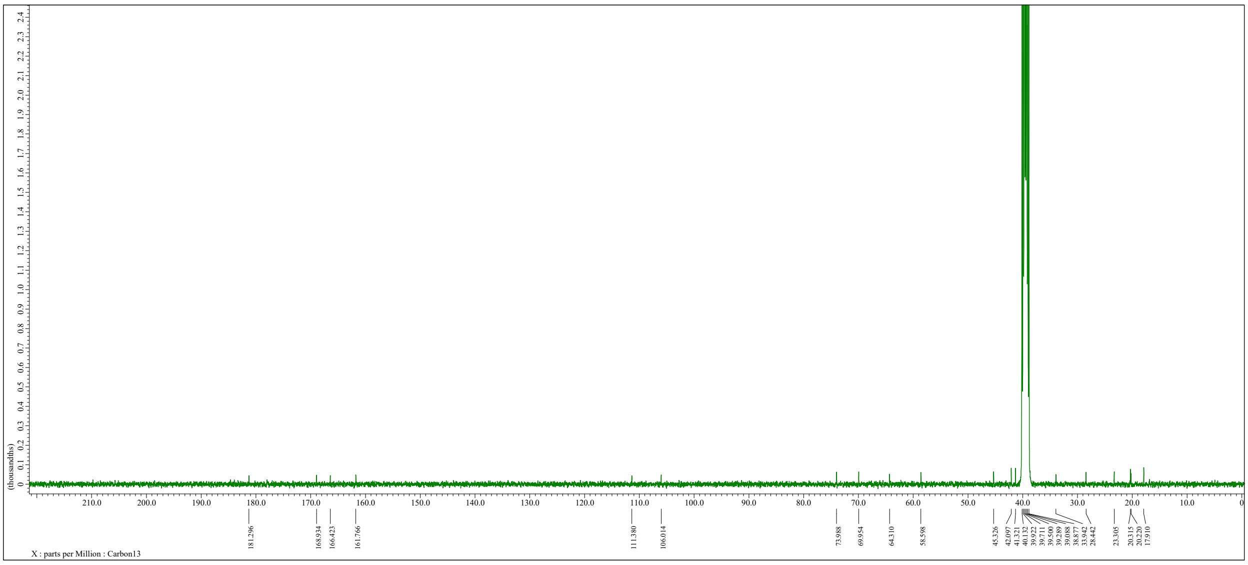

Figure S1.2. ^{13}C-NMR (DMSO-d_6, 100 MHz) spectrum of **2**.

2. Spectra of new compound 4:

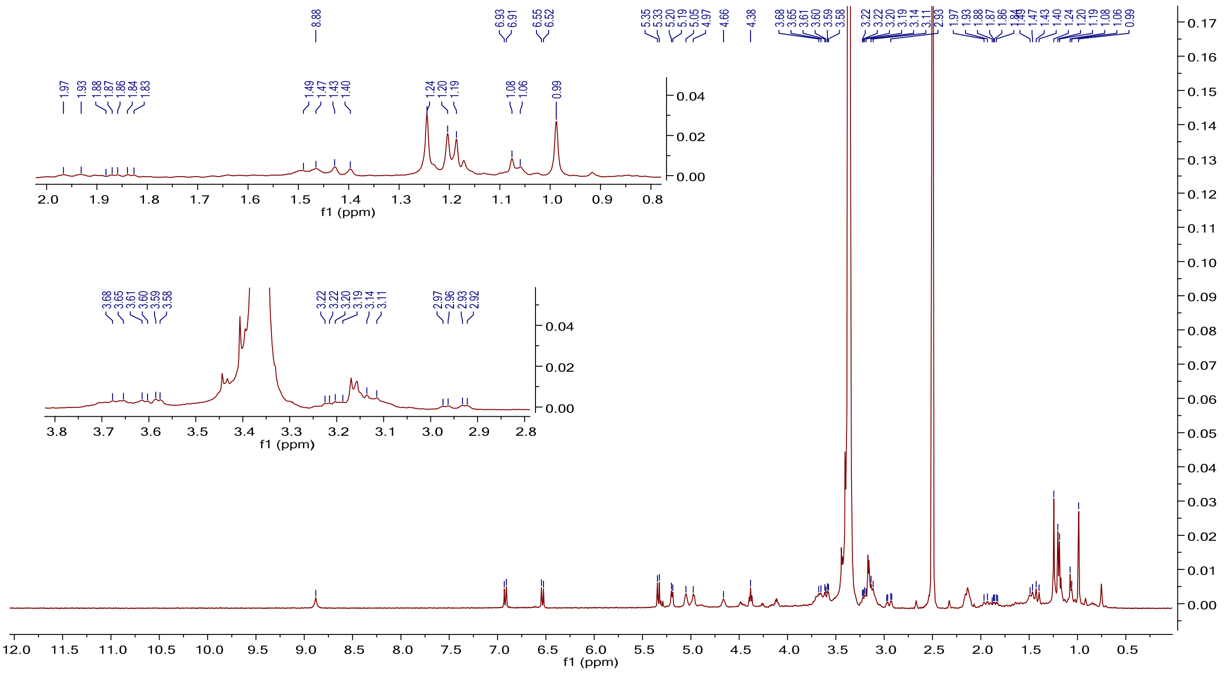

Figure S2.1. ^{1}H-NMR (DMSO-*d*$_6$, 400 MHz) spectrum of **4**.

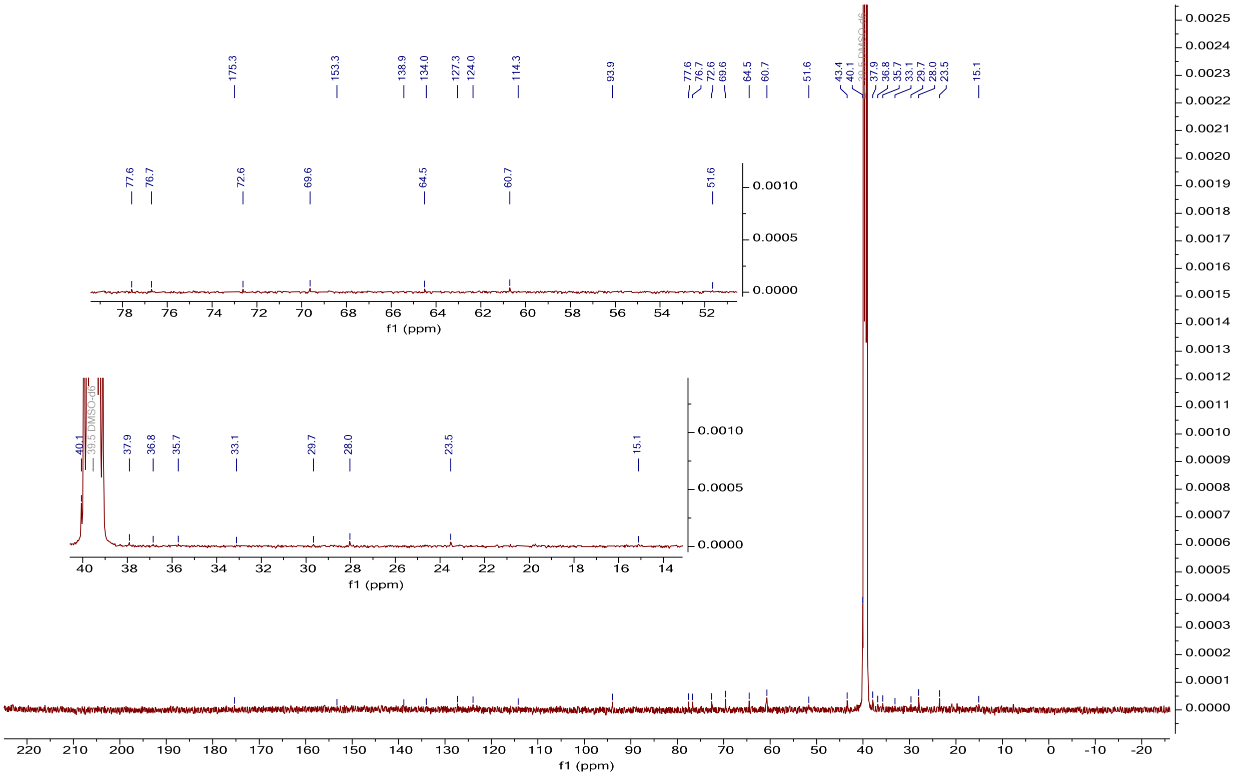

Figure S2.2. ^{13}C-NMR (DMSO-*d*$_6$, 100 MHz) spectrum of **4**.

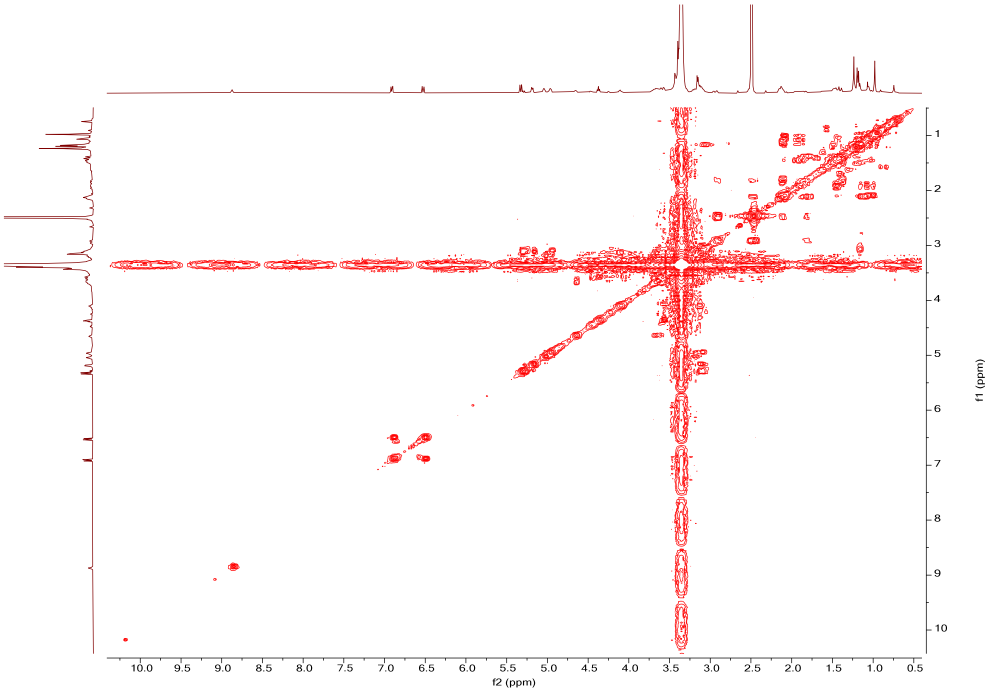

Figure S2.3. ^{1}H–^{1}H COSY spectrum of **4**.

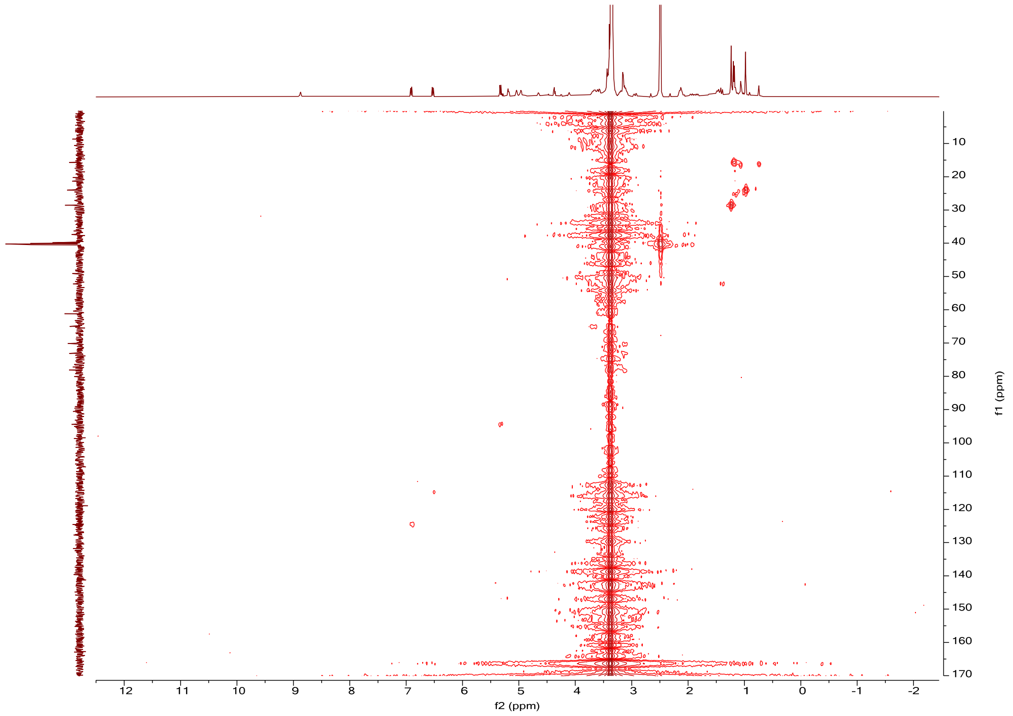

Figure S2.4. ^{1}H–^{13}C HMQC spectrum of **4**.

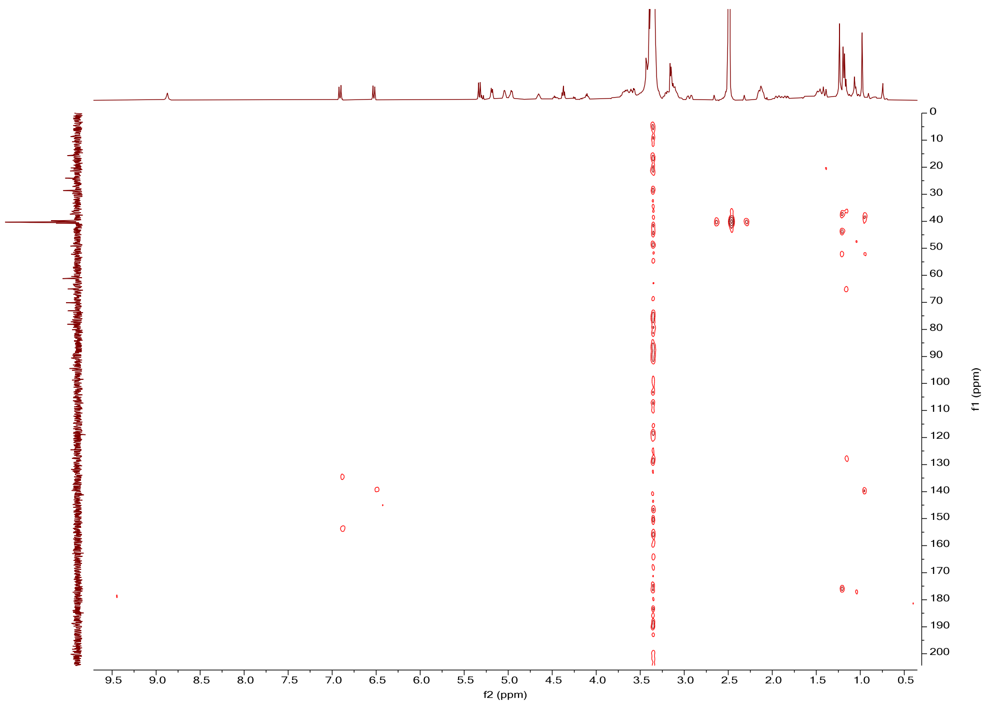

Figure S2.5. ^{1}H–^{13}C HMBC spectrum of **4**.

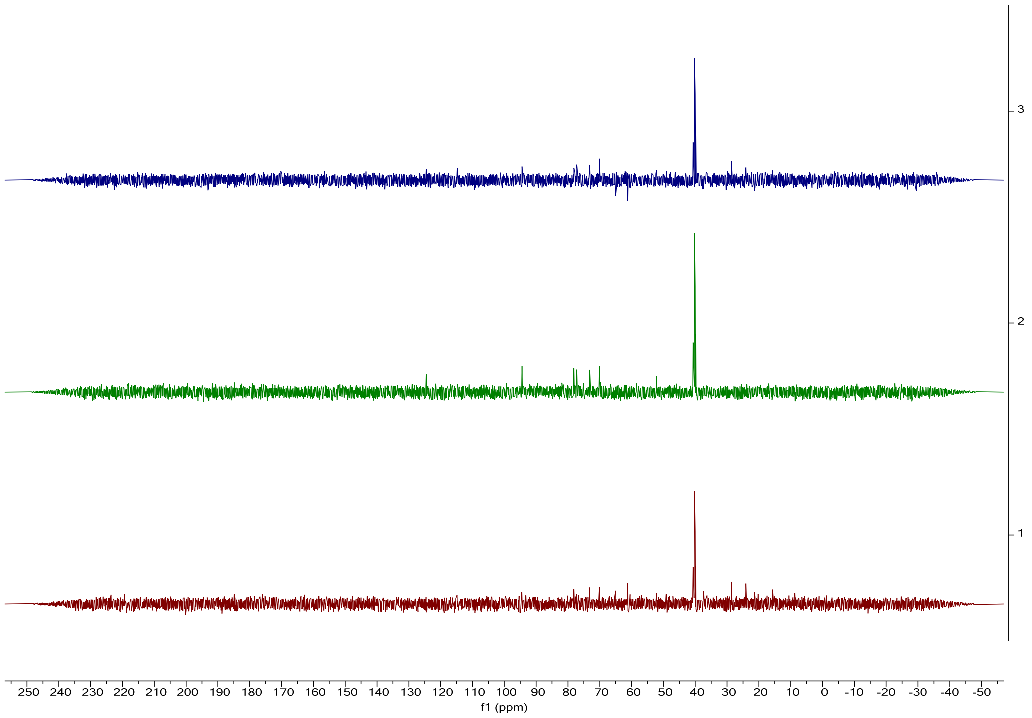

Figure S2.6. DEPT Spectrum of **4**.

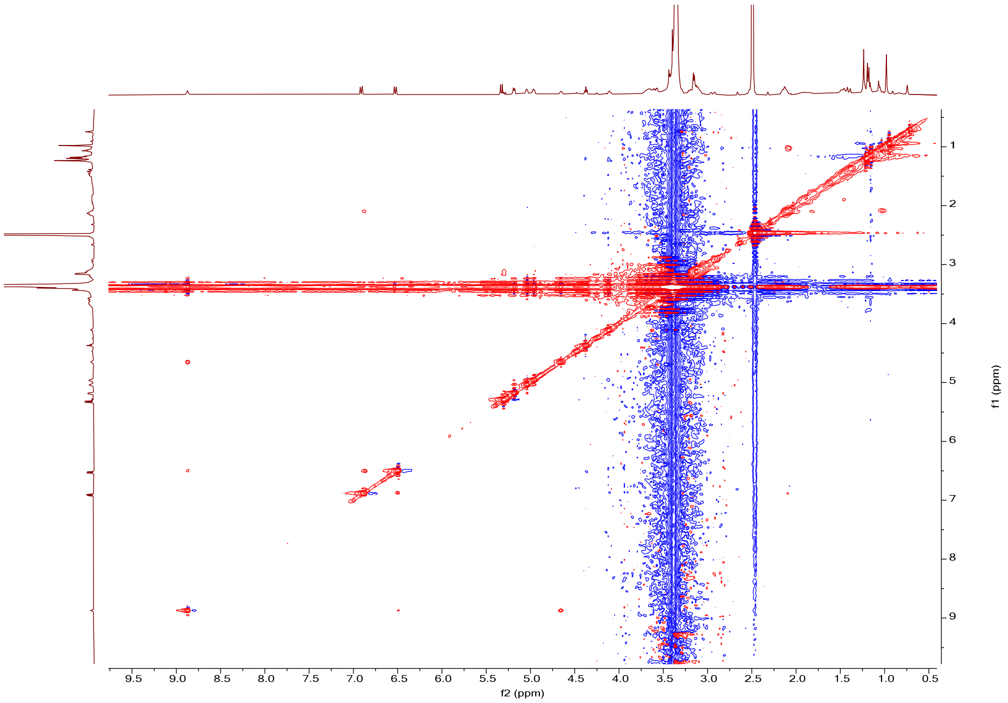

Figure S2. 7. ^{1}H–^{1}H NOESY spectrum of **4**.

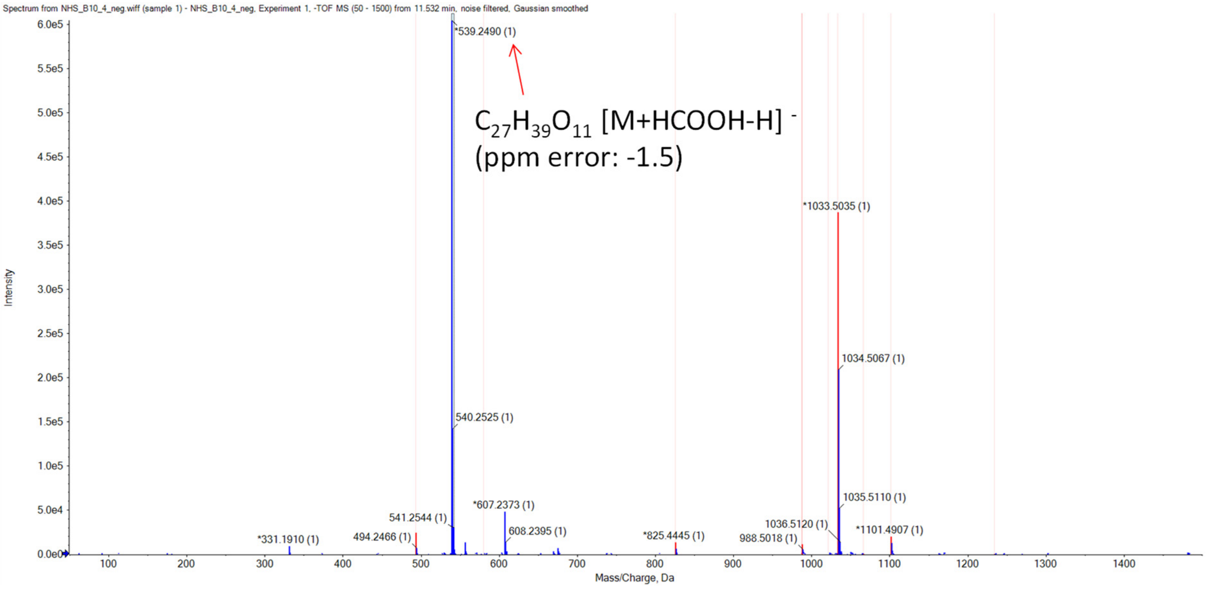

Figure S2.8. HR-ESI-MS spectrometry of **4**.

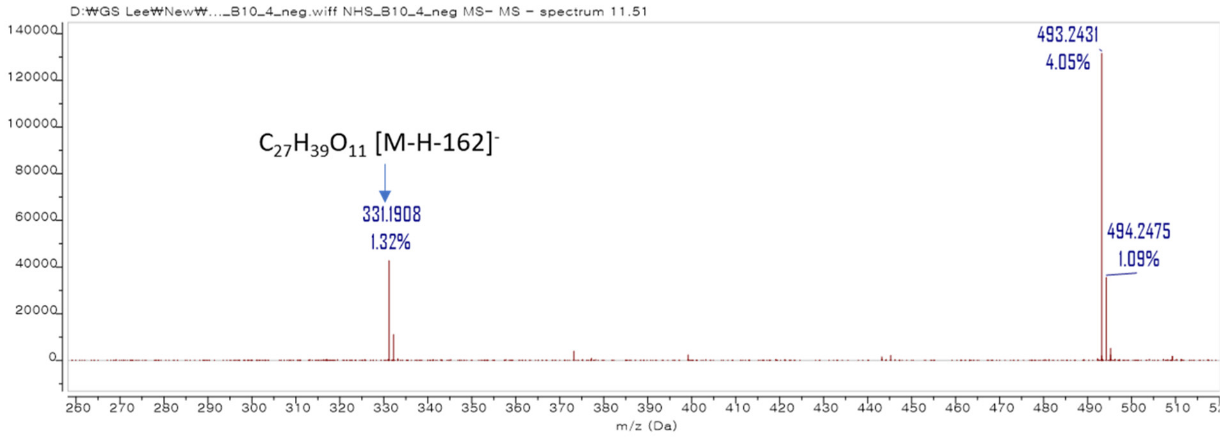

Figure S2. 9. Extend ESI-MS/MS spectrometry of **4.**
